# Fast Blob and Air Line Defects Detection for High Speed Glass Tube Production Lines

**DOI:** 10.3390/jimaging7110223

**Published:** 2021-10-25

**Authors:** Gabriele Antonio De Vitis, Antonio Di Tecco, Pierfrancesco Foglia, Cosimo Antonio Prete

**Affiliations:** Dipartimento di Ingegneria dell’Informazione, Università di Pisa, Largo L. Lazzarino 2, 56100 Pisa, Italy; gabrieleantonio.devitis@ing.unipi.it (G.A.D.V.); antonio.ditecco@phd.unipi.it (A.D.T.); antonio.prete@unipi.it (C.A.P.)

**Keywords:** pharmaceutical glass tube, image processing, defect detection, inspection systems, real time inspection

## Abstract

During the production of pharmaceutical glass tubes, a machine-vision based inspection system can be utilized to perform the high-quality check required by the process. The necessity to improve detection accuracy, and increase production speed determines the need for fast solutions for defects detection. Solutions proposed in literature cannot be efficiently exploited due to specific factors that characterize the production process. In this work, we have derived an algorithm that does not change the detection quality compared to state-of-the-art proposals, but does determine a drastic reduction in the processing time. The algorithm utilizes an adaptive threshold based on the Sigma Rule to detect blobs, and applies a threshold to the variation of luminous intensity along a row to detect air lines. These solutions limit the detection effects due to the tube’s curvature, and rotation and vibration of the tube, which characterize glass tube production. The algorithm has been compared with state-of-the-art solutions. The results demonstrate that, with the algorithm proposed, the processing time of the detection phase is reduced by 86%, with an increase in throughput of 268%, achieving greater accuracy in detection. Performance is further improved by adopting Region of Interest reduction techniques. Moreover, we have developed a tuning procedure to determine the algorithm’s parameters in the production batch change. We assessed the performance of the algorithm in a real environment using the “certification” functionality of the machine. Furthermore, we observed that out of 1000 discarded tubes, nine should not have been discarded and a further seven should have been discarded.

## 1. Introduction

Glass tubes, which are used for the production of pharmaceutical containers such as carpules, vials, and syringes, are typically made of borosilicate glass, since it is considered the most convenient solution for drug delivery systems and parenteral drug containers [1,2,3]. Borosilicate glass tubes can present defect-like flexible fragments called lamellae, lines of air and blobs (knot inclusions). Subsequent problems and pharmaceutical recalls may be a consequence of these defects [3,4,5,6,7,8] and indeed, glass issues have resulted in the withdrawal from the market of hundreds of millions of units of drugs packaged in vials or syringes [9,10,11,12]. Therefore, pharmaceutical tube manufacturers must check the quality of the tube produced, to avoid the fact that the finished product’s quality is affected by defects. Quality checks can be executed by an automated machine vision-based inspection system. The glass tube is, in fact, translucent and monochromatic, and defects can be detected by image processing algorithms, since they appear as changes in the gray level of images [13].

The Image Acquisition Subsystem and the Host Computer are the main components of a machine vision-based inspection system [13,14,15,16,17,18] for pharmaceutical glass tubes. The Image Acquisition Subsystem consists of a line scan camera, an LED illuminator, and a frame grabber. It captures digitized images (frames) and transfers them to the Host Computer’s memory. The Host Computer identifies the Region of Interest (ROI), i.e., the portion of the acquired image that only contains the object to be analyzed, eliminating other areas whose analysis is useless and would waste time in the subsequent stages, detects and classifies defects in the Defect Detection and Classification Subsystem, and sends discard commands to a Cutting and Discarding Machine.

The Image Acquisition Subsystem sets the quality level based on the production speed, and imposes time constraints on the system. This subsystem, in fact, generates images at a rate determined by the lines that constitute a frame and by the linear camera sampling rate. The Defect Detection and Classification Subsystem have to sustain this rate, to avoid loss of decisions. Therefore, the processing time needed for defect detection and classification is bound by the sampling rate of the linear camera. Advances in glass production processes require systems with faster production lines (from 5 m/s up to 10 m/s and over) and high accuracy in defects detection. Linear scan cameras with a higher sampling rate must be used to ensure that detection accuracy is kept constant or increased when the production speed accelerates. Consequently, defect detection and classification must be performed in a shorter processing time.

State-of-the-art implementations of glass tube inspection systems essentially rely on Canny’s algorithm [19] to perform defect detection. These solutions prove to be computationally expensive and inadequate to the performance requirements of advanced production lines. The objective of this work is to identify more efficient defect detection algorithms that allow the meeting of the timing requirements imposed by innovations in glass tube production lines.

Numerous industrial processes have adopted machine vision-based inspection systems [20,21,22,23,24,25,26,27], in addition to the related application domain such as the glass tube processing industry. In particular, the production of vials, bottles, and glasses [13,21,28,29,30,31]. However, specific requirements and factors characterize glass inspection. In particular, the glass tube has a cylindrical shape and, as a result of the production process, it is suspended. Moreover, it partially rotates and vibrates, and does not always result as centered with the illuminator. The cylindrical shape of the tube produces a sharply luminous intensity that decreases near the edges of the tube image, and a non-symmetrical luminous intensity when the illuminator is not centered. This makes the determination of the ROI complex possible, and prevents the use of some classic methods of defect detection. State-of-the-art solutions which adopt local thresholds [32] or edge detection [33] cannot satisfy the timing requirement of the system, while a global threshold [34] is inadequate due to the circular and irregular profile of tubes.

In this work, we propose a defect detection algorithm that does not change the detection quality compared to state-of-the-art proposals, but does drastically decrease the frame processing time. The algorithm performs row level processing to detect air lines, and column level processing to detect blob defects. In particular, working at the column level, we apply an adaptive threshold (one for each column) based on the Sigma Rule [35] to detect blobs. At the row level, we apply a threshold to the variation of luminous intensity along a row. Thereby, we limit the effects on detection of the curvature of the tube as well as tube rotation and vibration. The algorithm has been compared to state-of-the-art solutions in terms of detection quality and performance. The results point out that the detection phase processing time is reduced by at least 86%, with a throughput increase of at least 268%, achieving greater accuracy in detection. Further performance improvement can be achieved with ROI reduction techniques. Results show that the algorithm is suitable for a faster production process. Moreover, we have developed a tuning procedure to determine the algorithm’s parameter during the change of production batch. The performance has been verified in real operating conditions, and we have discussed implementation issues, by considering specific production-related problems. A first step towards the development of the algorithm that appeared in [36] is only devoted to blob detection, with partial evaluation and without tuning.

The work is organized as follows: Related works are presented in Section 2, while the implementation of an inspection system for pharmaceutical glass tubes is discussed in Section 3. This section highlights the most relevant requirements on defects and performance. The proposed algorithm is presented in Section 4, together with the tuning procedure. Section 5 is devoted to the setup and discussion of the experimental results, and also presents the performance assessment in a real world implementation. In Section 6, discussions on implementation issues are given, while Section 7 concludes the work.

## 2. Related Works

### 2.1. Related Inspection Systems

In literature, Foglia et al. [14] present the complete design of a glass tube inspection system. The image acquisition system is based on LED illuminators, CCD line scan cameras, and frame grabbers. The layout of lighting is in backlight configuration, which is the most used for the inspection of transparent parts [37]. The illuminator is set at a red wavelength, as the CCD sensors have the best sensitivity at this wavelength. The defect detection system is based on Canny’s algorithm [19], and functions properly up to a maximum tube speed of 4 m/s. Moreover, the authors propose a specific method to extract the ROI based on projections.

Solutions dedicated to the glass tube inspection are implemented by manufacturers (for example [38,39,40]), but they are not sufficiently documented (for commercial reasons).

A related application domain is the glass tube processing industry. In particular, the production of vials, which represents one of the final products obtained from the processing of glass tubes, and the related inspection systems. The inspection of vials consists of different tasks: Finding dimensional defects and finding defects in the vials. The last task is performed by analyzing different inspection zones: The mouth, the body, and the bottom [31]. In particular, the analysis of the body is relevant, since its structure is the same as the glass tube, and the adopted solutions can give hints to solve our detection problem.

Eshkevari, Rezaee et al. [28] present a system for automatic detection of dimensional defects in glass vials. The image acquisition system is based on CCD cameras and LED illuminators in backlight configuration. In addition, image acquisition is performed in two steps to obtain lateral and top views on the vials. ROI extraction is performed by applying a fixed (heuristically tuned) threshold to the images, and by elaborating the horizontal and vertical projections of the images (i.e., the sum of the luminous intensity of the pixels along a direction). To highlight the borders of the vials, the authors apply a threshold to the difference between the luminous intensity of two consecutive pixels and the previous ones, by starting from the four sides of the image frames and moving to the opposite direction. They present the results on a dataset, but do not show the performance achieved in a real working environment.

As for the detection of defects in glass vials, Yang and Bai [29] present a vial mouth defect detection system. Illumination is furnished by an annular LED light source, and a CCD camera is used to get the images. As for software architecture, a median filter is applied to reduce the image noise, and the border of the vials (the ROI) are individuated mainly by applying Canny. The defects are detected by applying a threshold algorithm to the polar coordinate version of the image. The paper mainly deals with software architectures and algorithms, and no data are given on real time performance of the system. The authors present the results on a dataset, but do not show the performance achieved in a real working environment, and thresholds are heuristically determined.

Liu et al. [30] describe a system for the analysis of the vial’s body and cap. The illumination is multi-angle [37] for cap defect detection and backlight for body analysis. As there are only small mechanical vibrations in the vertical direction (the transportation direction of vials in the apparatus is horizontal), the upper and lower boundaries of ROI are determined by the vial’s cap/body’s height in the acquired image. Then, the left and right boundaries of the ROI are obtained by finding the first edge in the image from the left or right, respectively. For the body, the left and right boundaries are located by taking a rectangle that has an equal distance from the edge of the body. To detect crack defects on the caps, the authors propose a method based on horizontal intercept projection. In addition, thresholds on the mean and standard deviation on the projections permit individuating defective caps. Nevertheless, the method does not permit locating defects and obtaining their geometric features. For the detection of body defects, a Gaussian filter is first applied to reduce noise. Then, a black top-hat transforms [41] to highlight the crack area, and a fixed threshold (derived from testing) is used to binarize the image. Six features on the resulting connected regions are evaluated to determine if the vial’s body is defective. This shows that the performance achieved by a dataset of 100 images is compatible with the production requirements.

Huang et al. [31] present a more general system for inspecting glass bottles, and present different algorithms for defect detection located on the mouth, the bottom, and the body of the bottle. The illumination system is in backlight configuration for the detection of body defects and the image size is 640 × 480 pixels. For ROI detection, they combined edge points with the histogram to guarantee the positioning accuracy. Defect detection utilizes the OTSU [34] algorithm, and features are determined on the connected region to classify a defective bottle. The authors perform the experiments on a test platform, designed to simulate the actual production situation, which show a detection rate of 99.95%, with a line speed of one bottle per second.

Moreover, inspection systems based on machine vision are used in other glass manufacturing sectors, where the appearance of defects, such as bubbles, lards, and optical distortion, cannot be avoided and might depress the glass quality. A real-time defect detection system for float glass fabrication has been proposed by Peng [13]. The system is composed of eight cameras and LED illuminators to cover the whole glass surface and achieve the required resolution. The acquired images present the stripes noise that is removed by building a benchmark image and applying a threshold to the difference between the reference image and the current ones. Then, the OTSU algorithm is applied to highlight the defects. The system has been tested in a float glass manufacturing plant, achieving a detection rate that is higher than 99%.

Adamo et al. [21] propose an online inspection system that is capable of detecting and classifying the typical defects (scratches and spots) of the surface of satin glass sheets. The authors present the design of motion system, whose speed is programmable to adapt it to the image acquisition unit. The lighting system is composed of four low-cost dark field linear sources, designed to guarantee a grazing light on the glass surface, that permit the correct visualization of irregularities. In the prototype, the image acquisition system is composed of two cameras based on a 1.3 Mpixels CMOS sensor. A manual system for camera and lighting system calibration is used. A mosaicking algorithm restores the image of the whole sheet starting from the partial images obtained with different cameras. The ROI is selected via the heuristic threshold. Defect detection is performed via the Canny algorithm. In addition, a procedure for tuning the thresholds, based on the analysis of the gradient magnitude for a defect free and defective image is proposed. The system has been successfully tested on glass sheets and on batches of sample images.

For the systems devoted to glass analysis, the main issue is the large dimension of the part to be inspected. The proposed solutions consist of using multiple cameras to capture the product image, and the processing techniques adopted can meet the timing requirements as they are not strict. In systems for the analysis of vials and bottles, either small-size images are used [28,31], or when employing high-pixel-count cameras, time requirements are less stringent [30,42]. This allows the application of algorithms that are computationally more complex than in the case of glass tube inspection, which is characterized by high process speed and high resolution. In all of the analyzed systems, however, it must be said that the production speed can be adapted to the inspection system, as stated in [21]. However, this is not possible in the production of the glass tube, where variations in the process speed produce changes in the physical properties of the produced tube.

### 2.2. Algorithms

Glass tube inspection can be carried out in three consecutive stages, as in other production environments [16,20,43]:

- Pre-processing stage;
- Defects detection stage;
- Defects classification stage.

The purpose of the pre-processing stage is to speed up the subsequent processing phases, identify and eliminate regions that should not be analyzed, and reduce errors introduced by image acquisition. Algorithms for contrast enrichment are usually implemented in this phase, as well as algorithms for noise reduction [44], and for detection and extraction of Region of Interest (ROI) [15].

The problem of ROI detection and extraction depends on the properties of the inspection object and the background, as well as the illumination system. When the background has a different light intensity (darker as in [21] or brighter as in [28]), threshold-based algorithms can be used to detect the ROI without altering its content. The detection phase can be followed by rotations, with the goal of generating rectangular matrices [45] that are easier to analyze. In the case of glass vials [28], the ROI of image is identified using threshold algorithms and it is subsequently extracted by analyzing horizontal and vertical projections. Moreover, ROI detection can be performed through edge detection techniques, which identify points where there are sudden changes in luminous intensity, as shown in [30] for the analysis of body and cap vials, and in [31] for the analysis of bottle bodies.

**Figure 1:**
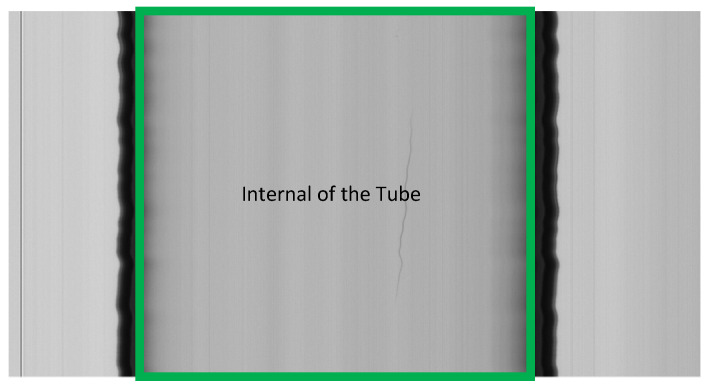
A tube image, where the region of interest (ROI) is highlighted or the internal of the tube. The frame is composed of 1000 × 2048 pixels, which corresponds to a tube length of 500 mm.

The ROI, for glass tube images, does not include the outer part of the tube and its edges. The edges are useless for detection since light rays are reflected off them and do not hit the camera sensor. Therefore, the ROI (Figure 1) consists of the inner part of tube (internal) within the frame. In this case, threshold techniques cannot be used to extract the ROI, since, due to the imperfect alignment between the camera and the tube, one of the areas outside the tube can be lighter and the other darker than the inner part of the tube. Edge detection techniques only allow detection on the outer edge of the tube, since, due to the curvature and vibration of the tube and the refraction of light, the areas between its edges have alternating light and dark zones, making edge detection difficult to apply. This effect has not been detected in inspection systems for vials and bottles [30,31] due to the lower resolution of the images used.

The ROI detection and extraction algorithm adopted in this work follows the approach presented in [14]. The projections of light intensity per column as averaged over the number of rows have the minimum values at the edges of the tube. The algorithm detects such minima, starting from the center of the tube and moving in opposite directions. A threshold is applied in the research, to avoid adopting local minima that is caused by defects or noise within the tube.

The defect detection phase is dedicated to identifying pixels that may belong to a defect within the image [16,20,43]. For this purpose, segmentation techniques [20] find wide application, and they can be implemented using edge detection [19,33,46] or threshold-based [13] algorithms.

In the classification phase, the segmented regions are analyzed by extracting and analyzing a set of attributes, possibly matching them with pre-defined defect classes [20].

In edge detection techniques, defects are detected by sharp changes in image brightness [33,46]. Canny’s algorithm [19] is considered as one of the best edge detection algorithms for analyzing noisy images [33]. In addition, it has been used for inspection and analysis in various manufacturing processes. For example, in [14] it has been applied for the inspection of glass tube, in [21] to satin glass, and in [22] to the wafer surface, in the analysis of bubble formation in co-fed gas-liquid flows [24], as well as in the analysis of ice shape on conductors [23]. However, the Canny algorithm uses time-consuming tasks such as: Applying a Gaussian filter for noise reduction, calculating the intensity gradient, and performing double-threshold comparisons. Therefore, the processing time required by Canny may not be compatible with the time constraints imposed by faster glass tube production lines.

Threshold-based techniques can use a single threshold for the whole image (global threshold techniques) or different thresholds for different regions of the image (local threshold techniques) [32].

The Otsu algorithm [34] is a global threshold algorithm, which produces effective image segmentation when the image luminosity histogram is characterized by a bimodal or multimodal distribution [20]. The threshold is chosen to maximize the variance among the classes of the image histogram [47]. Otsu’s algorithm is the basis of several inspection systems in the literature: In [20], it was used for surface inspection of transparent parts, in [13] and [25] for inspection during float glass fabrication.

**Figure 2:**
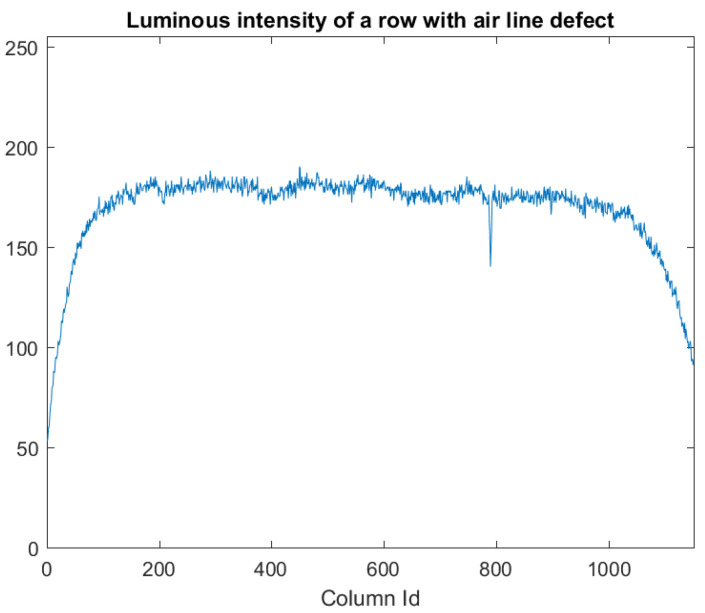
Luminous intensity for pixels that belong to a row with an air line defect.

The edges of the tube appear darker in a frame image due to the cylindrical shape of the glass tubes, and the pixels belonging or not belonging to a defect may have a similar luminous intensity (Figure 2). Consequently, the detection of defects can hardly be obtained with techniques based on constant thresholds (single or multiple) [36]. In addition, it is not possible to utilize algorithms that exploit a defect-free image or template, such as template matching or background subtraction techniques [13,48], as the tube vibrates and it does not have a perfectly circular shape (a shape similar to a “sausage”).

Niblack’s algorithm [32] is a segmentation technique based on local thresholds. It uses the mean value and variance of the luminous intensity in a window to analyze the pixel located in the center. The method is mainly applied in the analysis of documents, and allows the efficient separation of the background from the text [49]. It has been used in a vision system to observe the surface of the melt in the Ky method for the growth process of the sapphire crystal [27]. However, this method requires an update of the mean value and variance for each image pixel. In addition, the processing time may not be compatible with the real-time requirements of glass tube inspection.

As the processing time is a main constraint for the realization of real-time inspection of glass tubes, we consider a state-of-the-art implementation of the inspection system [14] to highlight how the three stages of inspection—(i) Image preprocessing; (ii) Defect detection; (iii) Defect classification—contribute to the total processing time. In this implementation, stage 1 is implemented through the ROI extraction algorithm described in [14]. Defect detection is implemented through Canny’s algorithm, while classification is implemented through the algorithm described in Section 4.4.

When analyzing the processing time required by the three phases of processing an image, the defect detection phase (Canny’s algorithm) is the one that requires the highest processing time (72% of the total time, 62.5 ms). This is due to the complexity of the algorithm, which performs time-consuming tasks such as applying the Gaussian filter, evaluating the intensity gradient, and applying a dual-threshold hysteresis algorithm. Canny’s algorithm finds application in the general case, and produces good results without considering the peculiarities of defects.

Since the defect detection phase takes most of the processing time, we derive a defect detection algorithm that achieves a shorter processing time by exploiting the characteristics of the most significant defects in the application domain.

## 3. Image Capture and Processing

**Figure 3:**
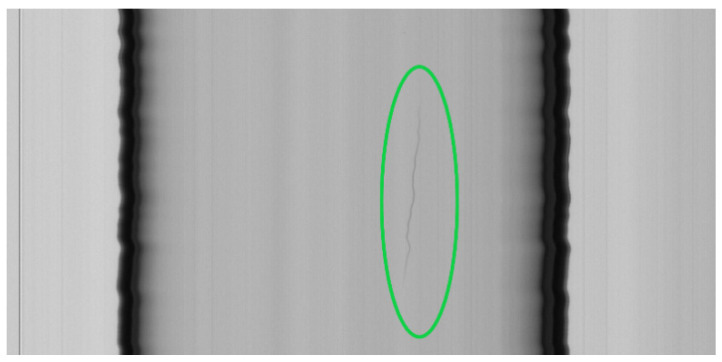
An air line defect (enclosed in a green oval). Due to the rotations and oscillations of the tube, the air line appears curved and irregular in the image, although it is straight.

**Figure 4:**
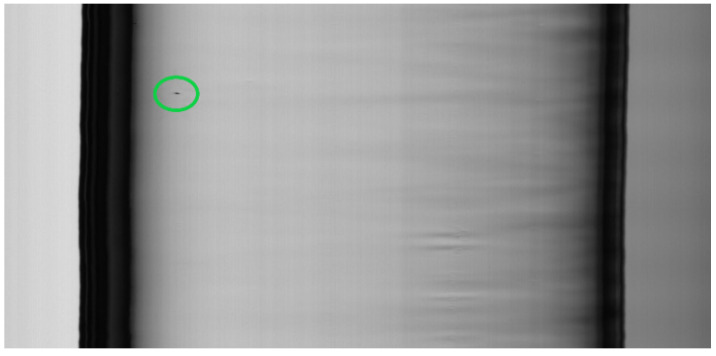
A blob defect (enclosed in a green oval). The tube has a more similar shape to a “sausage”, as can be seen on the center and right side.

The most critical defects in the production of glass tubes for pharmaceutical use [1,14], due to the significant effects on product quality and for their critical size are:

(1) Air lines. In the furnace, there are air bubbles that, when pulled by the drawing machine, generate air lines. In an image, they assume the form of darker lines of long dimensions (Figure 3). The final parts of the line are thinner than the central ones. The air lines become increasingly difficult to detect if they break (open lines) as they are thinner. 

(2) Blobs (inclusion of knots). Imperfections in raw materials in the furnace generate blobs. They have the shape of circular lenses on the surface of the tube, but appear on the captured image, due to the movement of the tube, as dark spots, orthogonal to the frame (Figure 4).

Tube slices that include these defects are not suitable for some applications. For example, tube slices that include air lines cannot be used for vaccine syringes, as they can cause a rupture of the inner surface of the syringe and contents may be contaminated. Vials cannot be produced from tube slices that include blobs, as they can easily give consumers the impression of low product quality.

The detection of these defects is realized by an inspection system based on machine vision [14], constituted by the Image Acquisition Subsystem and the Host Computer. The Image Acquisition Subsystem consists of a line scan camera, an LED illuminator, and a frame grabber. It captures digitized images (frames) and transfers them to the Host Computer’s memory. The individual lines captured by the line scan camera are grouped together by the frame grabber, generating a single frame. The 360-degree inspection of the tube is achieved with three cameras and the associated frame grabbers and illuminators.

The tube is inspected as it moves (web inspection [16]) from the furnace to the drawing machine. The Host Computer detects and classifies defects and, based on the results of the classification, sends discard commands to the Cutting and Discarding Machine, which cuts (and, if necessary, discards) the tube in slices of about 1.5 m. Communication among the modules is realized via an industrial distributed solution [50]. The algorithms for detection and classification are executed by the Defect Detection and Classification Subsystem. Discard decisions are taken based on discard policies, depending on the production order. Typical parameters are the number of defects in the tube slices (blobs and/or air lines), the maximum size of defects, and their (cumulative) length or area They can be set by operator via high usable interfaces [51].

### 3.1. Image Acquisition Settings and Requirements on Performance and Quality

A two-dimensional image frame is constructed by the frame grabber. A pixel in the image frame maps a virtual rectangle on the tube. The dimensions and area of the virtual rectangle are used to set the Image Acquisition Subsystem, which is based on the quality requirements of the system. Moreover, they are utilized to capture the size and area of defects detected in the frames [14]. The dimension of the virtual rectangle in the direction orthogonal to the tube movement (OrthogonalDimension) is derived from the optical magnification and the sensor size of the linear camera as in (1):

OrthogonalDimension = OpticalMagnification × SensorSize (1)

The dimension of the virtual rectangle in the direction longitudinal to the tube movement (LongitudinalDimension) is derived from the line acquisition period of the linear camera and the tube speed as in (2):

LongitudinalDimension = LineAcquisitionPeriod × TubeSpeed(2)

As for resolution requirements, the process demands a longitudinal resolution of 2 pixels per mm, while in our scenario the tube velocity varies, ranging between 0.5 and 4 m/s, depending on the tube diameter. The requirement on longitudinal resolution is satisfied with a value of 8 kHz for the line scan frequency of the linear camera. In fact, the longitudinal dimension has a value of about 0.5 mm, with a scan frequency of 8 kHz and at maximum tube speed, according to (2):

LongitudinalDimension = 1/8000 s × 4 m/s = 125 μs × 4 m/s = 0.5 mm

Regarding the other camera parameters, we used a CCD linear camera, with 2K CCD sensors and a sensor size of 7 × 7 μm, and an optical magnification of 1:2.15. The resulting orthogonal dimension is about 15 μm (1).

OrthogonalDimension = 2.15 × 7 μm = 15.05 μm

Therefore, one pixel maps an area on the surface of the tube of about 0.0075 mm^2^, with a height-to-base ratio of about 33.3.

As for the time requirements, the Host Computer receives images from the frame grabber at a rate that is equal to the line scan frequency of the linear camera, divided by the number of lines that make up a frame. Consequently, the period of acquisition of a frame depends on the acquisition period of a line and the number of lines that constitute a frame according to (3):

FrameAcquisitionPeriod = LineAcquisitionPeriod × NumberOfFrameLines (3)

Once a frame has been processed, the accept/discard command must be sent to the Cutting and Discarding Machine. A condition for executing the process in real-time is that the processing of a frame must be completed before the arrival of the next frame, i.e., (4):

FrameProcessingTime < FrameAcquisitionPeriod(4)

In this work, frames are composed of 1000 lines and the line acquisition period is 125 μs. The resulting acquisition period for each frame is equal to 125 ms (3). Therefore, the processing time is constrained to be less than this value (4).

The effect of the increasing tube speed, induced by advances in the manufacturing process, is a proportional increase in longitudinal dimension of the virtual rectangle (2), and thus a reduction in longitudinal resolution. If we want to hold the longitudinal resolution, it is then necessary to reduce the line acquisition period of the camera accordingly. However, the use of camera with a lower line acquisition period results in a reduction of the acquisition period for a frame (3), and consequently, the processing time of each frame must also be reduced (4).

### 3.2. Rational of the Proposal

The algorithm is intended to detect the following classes of defects:

- Air lines (Figure 3);
- Blobs (Figure 4).

As aforementioned, these defects can have a major impact on the quality of the produced tubes and are difficult to detect due to their features.

The algorithm works on the ROI derived, as in [14]. The projections of light intensity per column as averaged over the number of rows have minimum values at the edges of the tube. The algorithm detects the minima of the projections of luminous intensity per column as averaged over the number of rows, starting from the center of the tube and moving in opposite directions. A threshold is applied in the search, to avoid envisioning local minima caused by defects or noise within the tube. This approach minimizes the issues related to the rotation and vibration of the tube, and the imperfect alignment between the camera and tube.

**Figure 5:**
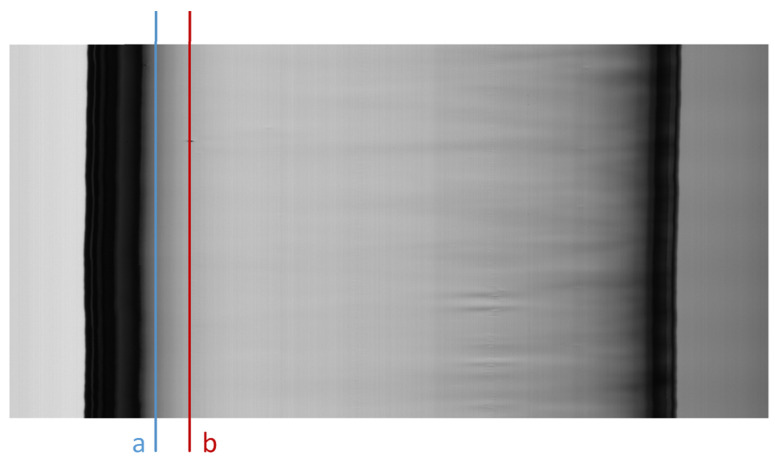
A frame with a blob defect. Column a is close to the edge of the tube and does not include a defect, column b includes a portion of a blob defect.

**Figure 6:**
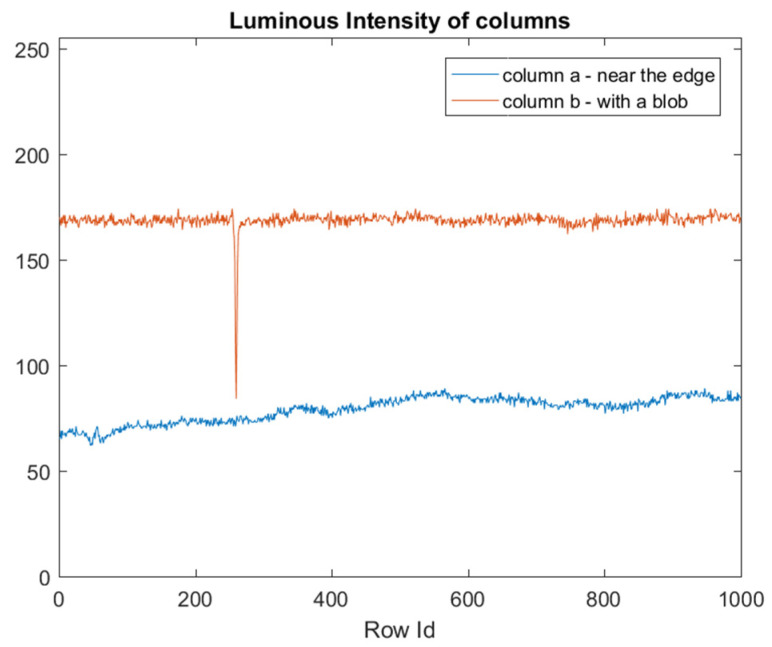
Luminous intensity of pixels that belong to columns a and b (which include a portion of a blob defect), as shown in Figure 5.

To detect the blob defect, we started by analyzing the luminous intensity of a column that includes this defect. Figure 5 represents a frame with a blob defect, and the luminous intensities of a column that include this defect is shown in Figure 6.

The effect of a defect is to lower the luminous intensity of pixels on which it is placed, compared to pixels on which it is not present. Consequently, defects could be detected via a threshold-based algorithm: Pixels with luminous intensity values below a threshold can be marked and passed to the classification stage.

**Figure 7:**
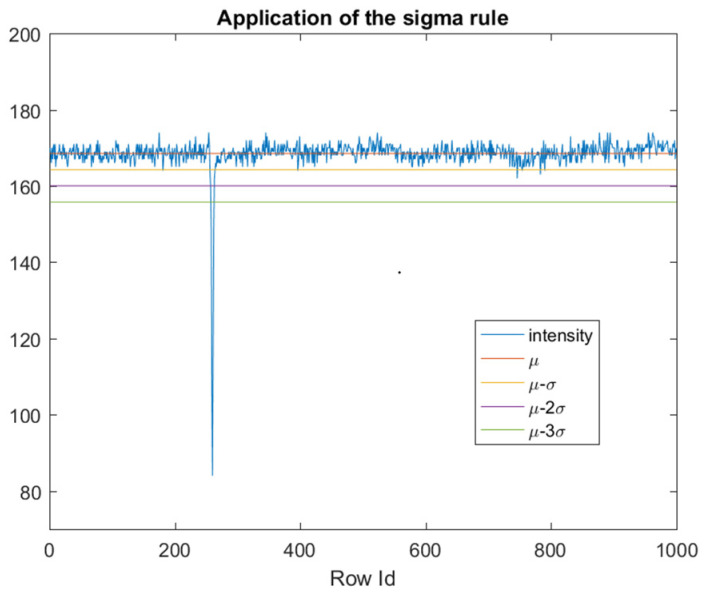
Application of sigma rule on a column that includes a blob defect. The mean of the luminous intensity of column b of Figure 5 is represented by line μ, while σ is the related standard deviation. Lines μ-σ, μ-2σ, μ-3σ, represent thresholds equal to μ minus 1, 2, 3 times σ ($k_{C}$).

Since the pixels that belong to columns that are close to the edge of the tube have a lower luminous intensity than the pixels of the blob (column a in Figure 5 and Figure 6), defects detection cannot be performed with a fixed global threshold. By considering that the mean luminosity of each column varies among columns, the threshold value must be adaptive to the mean luminosity of each column, and it must also take into account, due to image noise, the dispersion around the mean. In our idea, the Sigma Rule [35] can be utilized to determine the adaptive thresholds (one for each column). According to this approach, blob defects are detected via an adaptive threshold calculated as the mean value (μ) minus $k_{C}$ times the standard deviation (σ) of the luminous intensity of each column (Figure 7). We used this solution since, from the computational point of view, it is leaner than the other solutions, such as Median Absolute Deviation (MAD).

**Figure 8:**
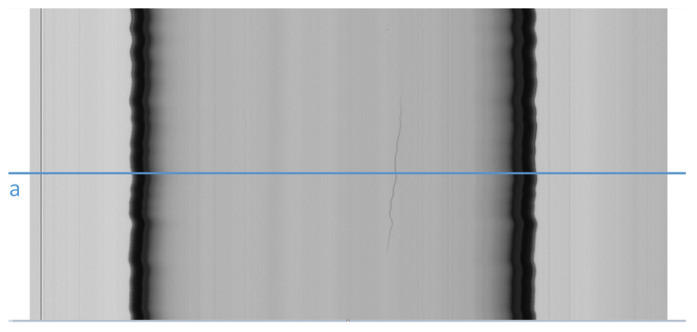
A frame with an air line defect. Row a is a row including the air line.

**Figure 9:**
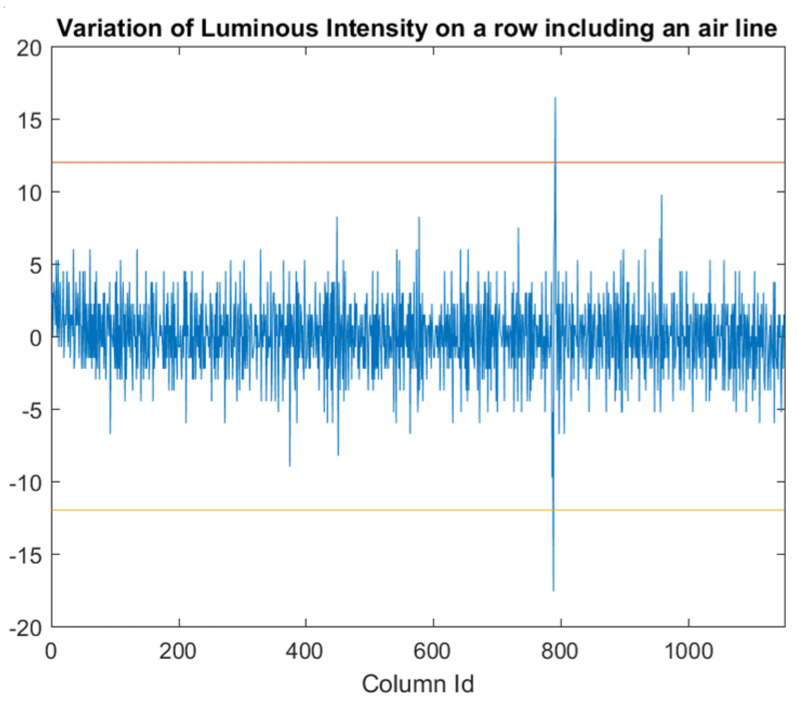
Variation of luminous intensity for pixels that belong to the same row with an air line defect inside the ROI. Values of negative peaks below (or positive peaks over) a given threshold may represent the air line defects.

To highlight air lines (Figure 8), from Figure 2 we can observe that, apart from low variations (noise) due to irregularities in the cylindrical shape of the tube, the luminous intensity varies gradually when moving along a row towards the border of the tubes, but suddenly changes in the correspondence of pixels belonging to an air line. Therefore, we calculate the variation of luminous intensity along the orthogonal direction of the tube for each row (Figure 9) to detect air lines. Peaks on this variation may indicate the presence of air lines. We assume from Figure 9 that the peaks belong to an air line defect, when the absolute value of these peaks is over a given threshold ($k_{R}$).

The use of the variation of luminous intensity is motivated by the significant change in luminous intensity near the air line (i.e., the absolute value of the peak over the threshold), due to the optical effect of refraction of light rays produced by the cavity of the air line. For this reason, this variation is greater than the variations induced by noise. Moreover, differently from the noisy points, the variation for the air line also appears in previous and subsequent rows. Therefore, during the classification phase, it is possible to individuate the air lines. In addition, the method presents a low computational cost.

## 4. The Sigma Algorithm

The proposed algorithm analyzes a glass tube image composed of grayscale pixels and only includes the ROI of tube image (source image). The luminous intensity of the image is represented with 8-bit values in the range [0, 255] with grayscale notation, and I(i,j) represents the luminous intensity of the pixel with coordinates i,j. The source image has the size of N rows and M columns. The pixels detected by the algorithm are written to a result image R(i,j), with the same dimensions as the source image. This image is initialized to 0 (black).

**Figure 10:**
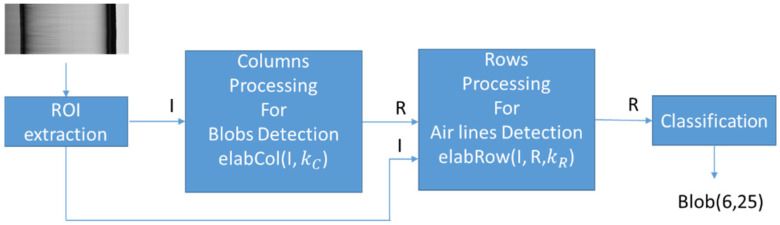
The two steps of the Sigma algorithm that, starting from the initial Image I, produce a result Image R that is used for classification. The $k_{C}$ and $k_{R}$ are the algorithm’s parameters. Classification generates a list of defects with their size (blobs and air lines).

The algorithm consists of two successive parts. The first part allows the identification of blob defects, while the second part allows the identification of air line defects. After that, the result image R(i,j) is ready to perform the classification (Figure 10).

### 4.1. Processing of Columns for Blobs Detection

1. For each column, the mean value m(i) of the luminous intensity and its standard deviation σ(i) are evaluated as follows:

$$m\left(i\right)=\frac{1}{N}\overset{N}{\underset{J=1}{\sum }}I\left(i,j\right)$$

$$\sigma \left(i\right)=\sqrt{\frac{1}{N}\overset{N}{\underset{j=1}{\sum }}I{\left(i,j\right)}^{2}-m{\left(i\right)}^{2}}$$

2. A pixel in column i is considered as belonging to an anomaly and the corresponding pixel is set to a white value (255) in the result Image R, if its luminous intensity is lower than m(i) minus $k_{C}$ times σ(i):

$$if\;I\left(i,j\right)<m\left(i\right)-k_{C}*\;\sigma \left(i\right)\;then\;R\left(i,j\right)=255$$

3. After processing each column, rows are processed to detect air lines.

### 4.2. Processing of Rows for Air Lines Detection

1. The finite difference along the horizontal direction is evaluated

D(i,j)=I(i+1,j)−I(i,j), ∀i∈{1…M−1}, ∀j∈{1…N}

2. The pixel (i,j) is considered as belonging to an anomaly, and the related pixel is set to a white value in the result Image R, if the absolute value of the finite difference is greater than a threshold $k_{R}$.

$$if\;\left|D\left(i,j\right)\right|\geq k_{R}\;then\;R\left(i,j\right)=255$$

3. After the processing of each row, the result Image R is ready for classification.

### 4.3. Algorithm

The pseudocode of the algorithm is shown in Algorithm 1. The acquired image is denoted as I. Other inputs are the constants ($k_{C}$ and $k_{R}$) and the R image. The algorithm returns an output image denoted as R (the result image). In the output image, defects are represented as white pixels (value of 255).

We do not report the implementation of some functions for simplicity, but an explanation is provided for each of them. For each column of the input matrix, the array of the standard deviation values is returned by function std_column(matrix), while the array of the mean values is returned by mean_column(matrix) abs(value), that calculates the absolute value of input value. Function ROI(ImageP) returns an image that only contains the ROI of the imageP parameter, by applying the algorithm described in [14].

The core of the algorithm is represented by two functions: The elabCol (row 1) and elabRow (row 16). Function elabCol(I,$\;k_{C}$) implements the processing of columns for blob detection, while function elabRow(I,$\;k_{R}$, R) implements the processing of rows for air lines detection.

The overall algorithm builds an Image I by applying function ROI to an acquired image (line 28). It calls (line 29) function elabCol(I,$\;k_{C}$) to perform columns elaboration and the returned image is stored in R (an image of the same size of I). In line 30, function elabRow(I,$\;k_{C}$, R) is called to perform row elaboration, and the result image is stored in R. This image is then passed to the classification stage.


**Algorithm 1** Proposed algorithm (Sigma).1. **function** elabCol (I, $k_{C}$)2.  N = Number Of Rows (I);3.  M = Number Of Columns (I);4.  m = mean_column (I);5.  s = std_column (I);6.  **for** (i = 1; i ≤ N; i++)7.   **for** (j = 1; j ≤ M; j++)8.      **if** (I(i,j) < m(j) −$\;k_{C}$*s(j))9.      **then** R(i,j) = 255;10.    **else** R(i,j) = 0;11.    **end if**12.   **end for**13.  **end for**14.  **return** R;15. **end function**16. **function** elabRow (I, $k_{R}$, R)17.  N = Number Of Rows (I);18.  M = Number Of Columns (I);19.  **for** (i = 1; i ≤ N; i++)20.   **for** (j = 2; j ≤ M; j++)21.    **if** (abs(I(i,j) − I(i,j − 1)) >$\;k_{R}$)22.    **then** R(i,j) = 255;23.    **end if**24.   **end for**25.  **end for**26.  **return** R;27. **end function** 28. I = ROI (acquired_image)29. R = elabCol (I, $k_{C}$)30. R = elabRow (I, $k_{R}$, R)


### 4.4. Classification

Defect classification is based on constructing a container of the detected pixels, defined as the smallest rectangle containing a set of adjacent white pixels, obtained in the defect detection step. Based on the defect analysis, the classification of blobs, air lines, and noise is performed by evaluating the characteristic features of the container.

For each container, we evaluate its area in pixel (A), and the ratio between the length and the height of the container (LHR) in terms of pixels number. We define the minimum area in pixel (A_Min_), the minimum value of ratio between the length and the height of a blob (BRLH_Min_), the maximum value of ratio between the length and the height of a blob (BRLH_Max_), and the minimum ratio (ALRLH_Min_) between the length and the height on an air line.

A potential defect is a container whose area is greater than the A_Min_ parameter. A blob is a potential defect whose ratio of the height to the width of the container is within the range [BRLH_Min_, BRLH_Max_]. An air line is a potential defect whose ratio of the height to the width of the container is greater than ALRLH_Min_.

As for the parameters, we utilize A_Min_ = 20 pixels, BRLH_Min_ = 0.1, BRLHR_Max_ = 0.8, ALRLH_Min_ = 2, the values are recommended by experts and are the consequence of the defect characteristics. As a result of the value assigned to A_Min_ and Equations (1) and (2), the smallest detectable defect has an area of 0.5 mm^2^.

### 4.5. Setting of $k_{R}$ and $k_{C}$ Values—Tuning

**Figure 11:**
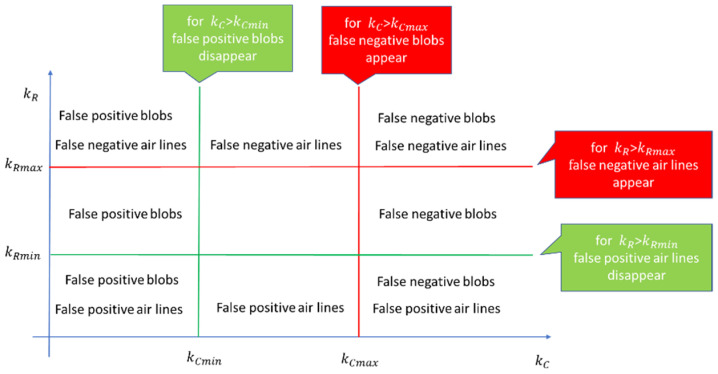
Scheme for identifying the parameters $k_{Cmin},\;k_{Cmax}$, $k_{Rmin}$, $k_{Rmax}\;$ for each frame in the tuning phase.

Real defects can be undetected (introducing false negatives) if $k_{C}$ and $k_{R}$ take high values. If $k_{C}$ and $k_{R}$ are too low, the algorithm detects as defects the containers that do not belong to any real defect (false positive). To determine the best $k_{C}$ and $k_{R}$ values, we developed a tuning procedure that, starting from a set of frames, determines the intervals of these values that do not introduce false negatives and minimize false positives (Figure 11).

In an industrial use of the inspection system, $k_{R}$ and $k_{C}$ depend on factors specific to the production batch (such as the thickness, size, diameter, dimension, color, and opacity of the glass tube). The tuning phase is performed on the changes in the production batch, to adapt parameters to the new production related factors. The procedure analyzes frames during the setup of the new production, one frame at each step.

The procedure (Figure 11) finds, for each frame, the $k_{Cmin}$ value which guarantees the absence of false positive blobs, and the $k_{Cmax}$ value below which there are no false negative blobs, as well as the $k_{Rmin}$ and $k_{Rmax}$ values which have similar meaning for the air lines.

The constants $k_{C}$ and $k_{R}$ can vary in the intervals [$k_{Cmin}$,$k_{Cmax}$] and [$k_{Rmin}$,$k_{Rmax}$] with the guarantee that: (i) No false positives are detected; (ii) real defects are detected in a defective frame (no false negative).

**Figure 12:**
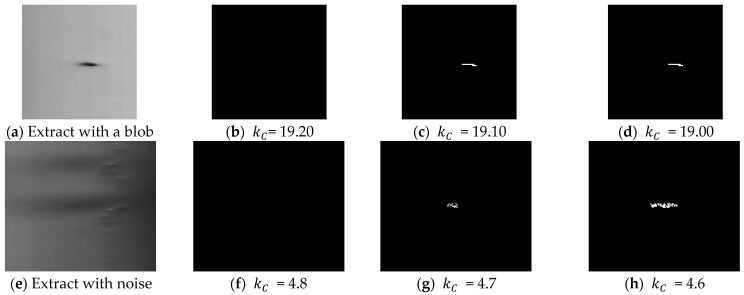
Different behaviors of a real blob (**a**) and a noisy image due to tube imperfection or noise (**e**) when changing the $k_{C}$ parameter. The blob in frame (**a**) is not detected for $k_{C}\;$ = 19.20 (**b**). It is detected if $k_{C}$ is decreased of a step of 0.1 (**c**). When $k_{C}$ is further decreased of the same step, the blob size is not changed (**d**). For image (**e**), there is no detection for $k_{C}$ = 4.8 (**f**), detection for $k_{C}$ = 4.7 (**g**). When decreasing $k_{C}$ of the same step, there is still detection, but the size of the blob is changed (**h**). The blob is a false positive.

The procedure is based on three experimental observations. Defects increase the variance of the columns on which they are located (observation 1). This rule allows us to compute the minimum values $k_{Cmin}$ and $k_{Rmin}$ that exclude the defects in a low variance zone. For blob defects, as glass has irregularities that produce a variation of luminosity intensity (the “sausage” effect as can be seen on the center and right side of Figure 4 and in Figure 12e), false positive blobs may also be in high variance zone. Nevertheless, if a real blob is detected for an assigned $k_{C}$ value, small decreases in $k_{C}$ determine small changes in the detected area. For false positive blobs (due to tube imperfections), small decreases in $k_{C}$ determine big changes in the detected area, as shown in Figure 12 (observation 2). For air lines, if $k_{R}$ values over $k_{Rmin}$ are present, they ensure that only real air lines are detected in high variance zone (observation 3).

Observations 1, 2, and 3 allow the algorithm to be executed in an unsupervised manner, without the need to manually label defects in the frame. If the frame does not include any defects, the procedure sets $k_{Cmax}=+\infty$, $k_{Rmax}=+\infty$.

Once the procedure has been applied to n frames, we obtain a set of n intervals [$k_{Cmin}\left(i\right)$,$\;k_{Cmax}\left(i\right)$] and [$k_{Rmin}\left(i\right)$, $k_{Rmax}\left(i\right)$], where i is an image in the set. We can evaluate the following intervals:

(5) $$[k_{Cmin},k_{Cmax}]\;\mathrm{where}\;k_{Cmin}=\max_{i=1}^{n}\left(k_{Cmin}\left(i\right)\right),\;k_{Cmax}=\min_{i=1}^{n}\left(k_{Cmax}\left(i\right)\right)$$

(6) $$[k_{Rmin},k_{Rmax}]\;\mathrm{where}\;k_{Rmin}=\max_{i=1}^{n}\left(k_{Rmin}\left(i\right)\right),\;k_{Rmax}=\min_{i=1}^{n}\left(k_{Rmax}\left(i\right)\right)$$

Parameters $k_{C}$ and $k_{r}$ can be chosen in these intervals.

**Figure 13:**
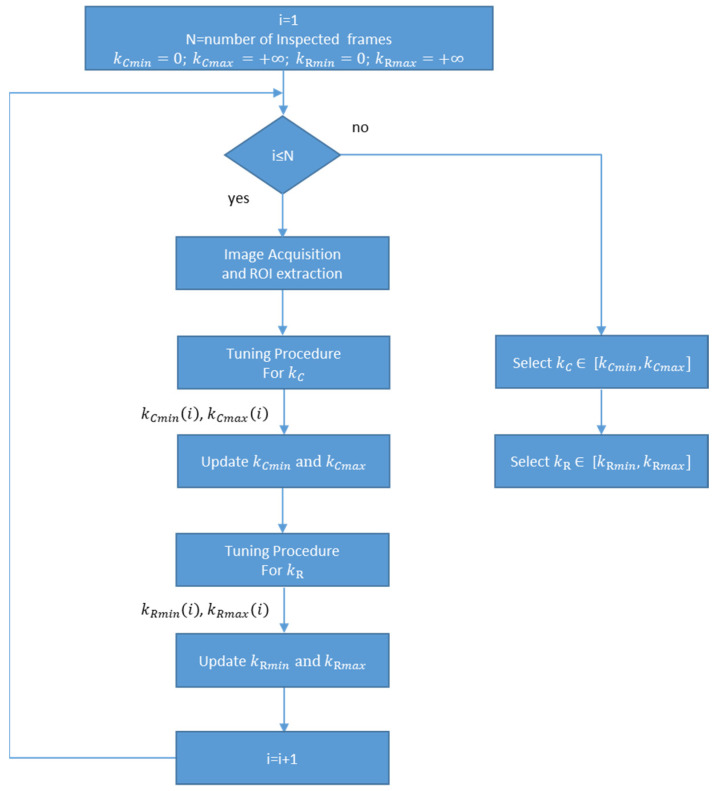
Flowchart of the tuning procedure. N frames are inspected, and the tuning procedure for $k_{C}$ and $k_{R}$ parameters are executed for each frame.

Figure 13 shows the flowchart of the tuning procedure. Here, N frames are inspected, and the tuning procedure for $k_{C}$ and $k_{R}$ parameters are executed for each frame.

The pseudocode of tuning procedure for the $k_{C}$ parameters is shown in Algorithm 2. After the frame acquisition and ROI extraction (line 1), the variance vector of the columns of frame V and its mean value m (line 2) are calculated. The value m+var_thre is utilized to separate high and low variance columns in line 7 (var_thre is set to 2 in our experiment, line 3); $k_{Cmin}$ is assigned 0; $k_{R}$ is assigned +∞; step defines the amount of increase for $k_{C}$ value (set to 0.1) in the search; and mul is utilized to determine if there is a big/small increase in the size of detected defect (set to 12, line 3). The algorithm increases $k_{Cmin}$ until there are no defects in the low variance zone (lines 4–7). Thereafter, a first value of $k_{Cmin}$ is determined (line 8). If the last classification has detected no blobs, indicating that there are no blobs in the frame, thus $k_{Cmax}$ is set to +∞ (lines 9–10). If there are blobs, the algorithm must update $k_{Cmin}$ if false positive blobs are detected, and must determine $k_{Cmax}$, i.e., the highest value that reveals blob defects, if any. Otherwise, $k_{Cmax}$ is set to +∞ (lines 21–23). This is done in lines 11–20, starting from $k_{C}$ = $k_{Cmin}$ (line 11), where the algorithm iterates increasing $k_{C}$ (line 14), as well as detects defects and false positives through the size criterion (observation 2) (lines 15 and 16). If false positives are present, the value of $k_{Cmin}$ is updated to $k_{C}$+ step (lines 17–19), and the iteration ceases when the number of blobs (true positive blobs) changes, if at least one was detected in the iterations or no more false positives are present (in the case of a frame with no true positive blobs) (line 20). The algorithm ends returning $k_{Cmin}$ and $k_{Cmax}$.


**Algorithm 2** Tuning algorithm for the $k_{c}$ parameter.
 « A frame is acquired, and ROI is extracted » « Calculates vector V, the variance on the columns of the ROI and its mean value *m* » $k_{Cmin}$ = 0, $k_{R}$ = +∞, step = 0.1, var_thre = 2, mul = 12 do  $k_{Cmin}$ = $k_{Cmin}$ + step  « Perform detection and classification. Parameters of Sigma are ($k_{Cmin}$, $k_{R}$) » while « There is at most a blob with V (j) < *m*+ var_thre for each column j of the blob » //observation 1 // a first value of $k_{Cmin}$ is determined if «There are no blobs with the last classification » then $k_{Cmax}$ = +∞ else $k_{C}$ = $k_{Cmin}$  « Determine the list of blobs »  do   $k_{C}$ = $k_{C}+$ step   « Perform detection and classification. Parameters of Sigma are ($k_{C}$, $k_{R}$) »   « Determine the list of blobs and label as false positives the blobs that have changed the size of at least mul * step times since the previous step » // observation 2   if « The number of false positives » ! = 0    then $k_{Cmin}$ = $k_{C}+$ step   endif  while « The number of blobs is the same as in the previous step and is not zero» OR « there are only false positive »  if « At least a blob has been detected in the previous iteration»  then $k_{Cmax}$ = $k_{C}-$ step  else $k_{Cmax}$ = +∞  endif endif return ($k_{Cmin}$, $k_{Cmax}$)



The pseudocode of tuning procedure for the $k_{r}$ parameters is shown in Algorithm 3. After the frame acquisition and ROI extraction (line 1), the variance vector of the columns of frame V and its mean value m (line 2) are calculated. These steps are executed once if the two tuning procedures are executed together. Here, $k_{Rmin}$ is assigned 0; $k_{C}$ is assigned +∞; and step defines the amount of increase in the search for $k_{R}$ value (set to 1 in our experiment, line 3). The algorithm increases $k_{Rmin}$ until there are no defects in the low variance zone (lines 4–7). Thereafter, the value of $k_{Rmin}$ is determined (line 8). To evaluate $k_{Rmax}$, if the last classification has detected no air lines, $k_{Rmax}$ is set to +∞ (lines 9–10). If the air lines are classified in high variance zones, the algorithm starts from $k_{Rmax}$ = $k_{Rmin}$ and increases to the highest value that keeps the number of detected air lines constant (lines 11–17). The algorithm ends returning $k_{Rmin}$ and $k_{Rmax}$.


**Algorithm 3** Tuning algorithm for the $k_{r}$ parameter.
 « A frame is acquired, and ROI is extracted « Calculates vector V, the variance on the columns of the ROI and its mean value *m* » $k_{Rmin}$ = 0, $k_{C}$ = +∞, step = 1 do  $k_{Rmin}$ = $k_{Rmin}$ + step  « Perform detection and classification. Parameters of Sigma are ($k_{C}$, $k_{Rmin}$) » while « There is at most an air line with V (j) < *m*+2 for each column j of the air line » //observation 1 // $k_{Rmin}$ is determined if « There are no air lines with the last classification » then $k_{Rmax}\;$=+∞ else $k_{Rmax}\;$=$\;k_{Rmin}$  do   $k_{Rmax}$ = $k_{Rmax}$ + step   « Apply the Sigma algorithm and classification with $(k_{C}$, $k_{Rmax}$) »  while « The number of air lines is constant » // observation 3  $k_{Rmax}$ = $k_{Rmax}$ − step end if $return\;(k_{Rmin}$, $k_{Rmax}$)



## 5. Results

A prototype inspection system has been implemented in a real glass tube foundry. The main components of the Image Acquisition Subsystem are reported in Table 1. The illuminator provides a uniform, high-power light source to prevent changes in ambient lighting resulting in changes in the values captured by the linear camera. A PC equipped with an intel i7-940 CMP [52,53] deploys the Host Computer. The inspection algorithms have been implemented in OpenCV [15,54] in a task whose execution is triggered upon completion of the transfer of a frame into the main memory. Therefore, the processing of one frame is overlaid on the transfer of the next frame. The Host Computer communicates by means of a CIFX 50E-DP card [55] with the Cutting Machine. Since the distance between the Cutting Machine and the Host Computer is greater than 5 m, from the processing of a frame, 1 s is available to generate the command of discard. This time is superimposed on the processing of subsequent frames, and therefore was neglected in our evaluation.

In the implementation, the functionality of the verification and certification of production quality for the customer has been added. This functionality discards all of the tubes produced in a given time interval, and allows the certification of the machine data against the real control, which is carried out offline by expert operators.

The evaluation of the system was carried out in two ways. In the first, we used a dataset to compare the proposed solution with others existing in the literature, developing a system that emulates the architecture of the real system. Then, through the functionality of verification and quality certification, we evaluated the proposed solution in the real system.

### 5.1. Comparison with Other Solutions

These evaluations are made via a 30-frame dataset (each frame has a size of 2048 × 1000 pixels), captured in the production phase. In the dataset, one frame contains two air lines, another contains three blobs, while 10 frames include a blob, six frames include an air line, and 17 frames are defect free. A machine similar to the one used in production was used to carry out the tests.

In the evaluation, four metrics are considered: Processing time, type and number of detected defects, area in pixels of the blob, and length in pixels of air lines. In order to evaluate the processing time, the complete dataset was analyzed 400 times [59]. In each run, to reproduce real working conditions and interferences, the three CPU cores (each dedicated to the processing of frames coming from a camera) perform the processing on randomly selected frames preloaded in RAM in a task statically assigned to a core. At the end of the experiment, the maximum total processing time was measured, since it represents an estimate of the worst case execution time [59], and the average processing time of each processing step (ROI extraction, defect detection, defect classification) was measured.

Regarding the type and number of defects, we determined: (i) The number of true positives (TP), i.e., precisely classified defects; (ii) the number of false positives (FP), i.e., detected defects not corresponding to real defects; (iii) the number of false negatives (FN), i.e., actual defects not detected. Human experts determined the actual defects (expected value) by visually inspecting the tubes. These expected values constitute the ground truth.

We have compared the performance figures of Sigma to the performance figures achieved by the Canny algorithm [19] and the Niblack algorithm [32]. In all of the experiments, we have considered the version of the ROI identification algorithm proposed in [14], as the pre-processing phase, and the classification based on containers described in Section 5.4. After executing the tuning algorithm of Section 5.3, the resulting parameters of the Sigma algorithm were $k_{C}$ = 4.91 and $k_{R}$ = 12. Both Canny and Niblack parameters were obtained through a design space exploration with the goal of optimizing the detection quality. For the Canny algorithm, we obtained the parameters 35–80. A threshold K of −1.7 and a window of 20 × 20 pixels were obtained as Niblack’s parameters. Table 2 lists the algorithms utilized in the experiments for the various steps of detection and their parameters.

By analyzing the results related to the accuracy in defect detection (Table 3), the three algorithms detect all of the real defects in the tubes (the number of true positives is equal to the number of real defects) and do not present false negatives. The Sigma algorithm has the least number of false positives, while that number is high for Niblack. This behavior is due to darker horizontal spots resulting from the imperfectly circular shape of the tube. The use of a window, in Niblack, does not allow the separation of these spots from the defects. Moreover, the same considerations apply to the defective frames (Table 3). In particular, to the frames that the system should discard. We have introduced this further classification as a frame that can contain multiple defects.

**Figure 14:**
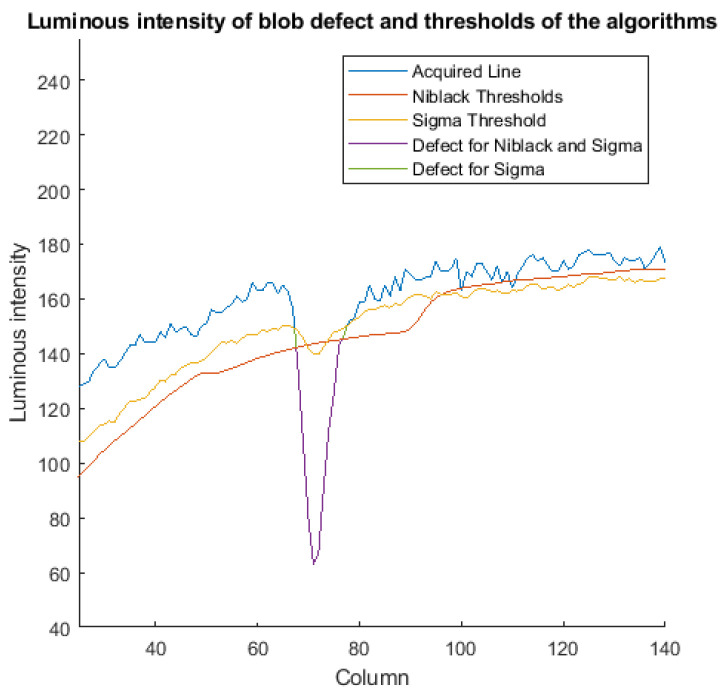
Luminous intensity (blue) of the portion of a row, including a blob defect and threshold used with Sigma (yellow) and Niblack (red). In correspondence of the blob columns, Niblack thresholds are lower than Sigma, and the resulting detected region (purple) is smaller than Sigma (green).

Table 4 and Table 5 show the area of the blobs and the length of the air lines (in pixels) for defects classified as true positives. As for the blobs area (Table 4), the Sigma algorithm has the best performance in terms of cumulative percentage and relative error (Avg Abs Error, which is the average of the absolute error). Canny and Niblack have the worst performances. At the end of the Canny algorithm, the dilate morphological operator is usually applied [15] to improve the detection of connected regions (which is useful in our case for air lines). However, the application of this operator introduces a high error in the detection of the blob area (83%). On the other hand, the thresholds generated by Niblack, tend to be lower than those of Sigma. In correspondence with the windows that enclose blobs (Figure 14), the resulting thresholds are less capable in the detection of the exact contours of the blob. In terms of air line lengths, the three algorithms perform similarly. The cumulative error in the three cases does not exceed 15%.

**Figure 15:**
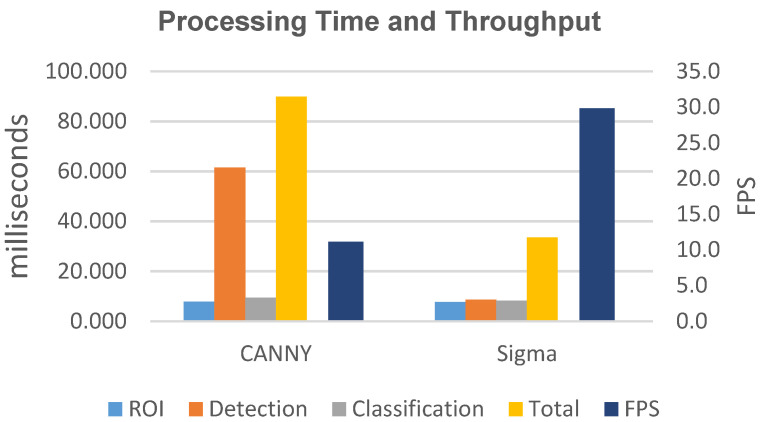
Performance data for the Canny and Sigma algorithms.

In terms of processing time (Table 6), Niblack presents the highest execution time and the minimum throughput. In addition, it is not compatible with the performance requirements of the Inspection System. In Figure 15, we only report the performance data for the Canny and Sigma algorithms. In particular, we report the average time for the extraction of the ROI (ROI), the average time of the defect detection algorithm (Detection), and the average time for the classification (Class). Moreover, we show the maximum among the total processing times for all of the frames, represented as Total, and the achievable throughput, represented as Frames Per Second (FPS). The Sigma algorithm has the best performance, with a reduction of the processing time of the algorithm (Detection) of 86% and of the total time (total) of 63% compared to Canny. The resulting throughput, for Sigma is 29.8 FPS, which is an increase of 268% compared to Canny.

The Sigma algorithm is then successful in decreasing both processing times for defect detection and total processing time, resulting in a similar or better quality of inspection than the algorithms considered in this work. In particular, with the adoption of the Sigma algorithm (Table 3, Table 4 and Table 5), the number of true positives is correct, while the number of false positives is lower than the other algorithms, and the accuracy in detecting the size of the defects is higher. This is all with an increase in throughput of at least 260%. The solution implementing the Niblack algorithm presents the worst detection quality, but also the worst performance in our problem.

According to (4), the reduced processing time obtained with Sigma makes it possible to increase the sampling rate of the linear cameras. Therefore, smaller defects can be detected due to the better resolution of the collected frames (2). Another implication of the reduction in processing time is that the quality of defect detection should not be changed by an increase in production speed (2).

To further reduce the processing time of the defect detection phase, the DSDRR technique can be applied [60]. This technique is a preprocessing technique which, via detrended standard deviation and double threshold hysteresis, further reduces the ROI, by eliminating regions where it is sure that defects are not present. The technique is independent from the defect detection algorithm (and it does not alter its quality of detection [60]). Therefore, it can be applied to both Sigma and Canny algorithms (we do not consider the Niblack algorithm in the evaluation due to its low performance and low detection quality).

The use of the DSDRR technique allows the reduction of the processing time for both algorithms (Table 7). In particular, a reduction is observed in the defect detection and classification. The throughput increase is three times for Canny and two times for Sigma (DSDRR is more effective for more complex algorithms). However, Sigma continues to be the most performing algorithm, with a throughput that is 1.73 times the throughput by Canny.

### 5.2. Performance and Quality Assessment in a Real World Implementation

The Sigma algorithm was implemented in the inspection system described at the beginning of the section. We utilize the functionality of verification and quality certification of the system to evaluate the quality parameters obtained by the algorithm in a real environment.

To highlight the limit of the algorithm, we performed a stress test, in which the system was made to work with very stringent discard parameters, i.e., the presence of a single defect on the tube determines the discarding of it. In real cases, it is the set of defects present on the tube and/or their size that determines the discard criterion.

In the stress test, all of the tubes checked by the machine were discharged, stored, and analyzed offline by expert operators. The experts analyze individual tubes using a black background and orthogonal white fluorescent light to highlight even the smallest defects.

To assess the quality of the system, the machine counts the following quantities: Tubes Accepted by the inspection system, Tubes Discarded by the inspection system, Tubes Validated by operators as actually meeting the quality requirements, Tubes Invalidated by operators actually not meeting the quality requirements, False positive Tubes (TubeFp), i.e., tubes discarded by the inspection system but meeting quality requirements and False negative Tubes (TubeFn), i.e., tubes accepted by the inspection system but not meeting the quality requirements. True positive Tubes (TubeTp) are tubes correctly discarded by the system. As metrics to assess the quality of the system, we utilize the Precision Rate, which identifies the effectiveness of the discard, and the Recall Rate which identifies the quality of the product after the discard phase [61,62]. The precision P is defined as:

(7) $$P=\frac{TubeTp}{TubeTp+TubeFp}=\frac{TubeInvalidated-TubeFn}{TubeDiscarded}$$

and recall R is defined as:

(8) $$R=\frac{TubeTp}{TubeTp+TubeFn}=\frac{TubeInvalidated-tubeFn}{TubeInvalidated}$$

This is due to the fact that

(9) TubeInvalidated=TubeTp+TubeFn

(10) TubeDiscarded=TubeTp+TubeFp

In an industrial scenario, the product quality rate should be as high as possible after inspection, which means adopting a strict inspection technology to minimize the number of false negatives. However, a strict inspection technology may result in a high number of false positives, which leads to low productivity and high cost in production. A good defect detection system should be characterized by high precision (P) and high recall (R).

We performed the stress test on two separate production batches, which differ in caliber, i.e., outer diameter and thickness. The test length was set to 300 tubes (to give operators a reasonable inspection time). Table 8 shows the result obtained.

The results show the good values we obtained for precision and recall. Moreover, it can be observed that the machine performs better in real conditions than what was obtained with the training set. This is due to the fact that the training set was selected with the images that make the detection more critical. The processing time observed during stress testing and the actual operation are in line with what was estimated using the datasets, confirming the validity of the estimates obtained. In addition, all of the frames processed in the test were scanned and classified in time. Finally, it must be said that the test was performed under stress conditions. In real supplies (with commercial criteria of quality currently accepted by pharmaceutical companies), R results are higher than 0.993 and P results are higher than 0.991. In practical terms, out of 1000 discarded tubes, nine should not have been discarded and a further seven should have been discarded.

## 6. Discussion and Implementation Issues

In the case of implementation in a real system, it is necessary to consider the case of a defect extending over more than one frame belonging to the same tube. To handle this situation, the classification of the defects of a frame takes place after the next frame has been processed (defect detection). For adjacent pixels present on the frame proximal to the bottom edge, adjacent pixels with the same coordinates are searched for on the top edge of the next frame. If these pixels exist, the two relative containers of the detected pixels are merged, and the potential defect is classified according to the algorithm described in Section 4.4.

Regarding the implementation of an inspection machine for a glass foundry, it requires the consideration of some production-related problems: Management of the phase of production caliber change, resetting of production in the case of tube breakage, and automatic drafting of the production report.

The management of the caliber change consists of aligning the machine with the characteristics of the new production. The tube rests on rollers, and passing from a large to a small caliber, the axis of the camera and the illuminator do not intercept the axis of the tube. In particular, the tube will be located in the non-uniform illumination area of the illuminator itself for two illuminators. In general, the solution consists of repositioning (manually or automatically) the group of cameras and illuminators to have the two intersecting axes. The hostile environment of the foundry and the need to make the machine simple and mobile prompted us to find a software solution. As was conducted in the Sigma algorithm, which considers the effects on the non-uniform illumination.

The tube is cooled by running 60–80 m on rollers in the presence of puffs of cooling air to the machine that pulls the tube. The tube can break on the way. An operator must pick up the tube from the start and quickly attach it to the pulling machine. All of the machines present on this route must be made in a way as to facilitate this operation. In particular, the inspection machine must have the mechanical structure that supports the cameras and illuminator that can be opened as a shell, in order to close it again when production is completely restored.

Tubes with many defects represent a cost for the tube transformation industry into carpules, vials, and syringes, since these pharmaceutical containers made with a defective tube will not pass the quality control. For this reason, the customer specifies the minimum quality that the tubes must have and tolerates the presence of a minimum part of tubes that do not comply with this specification.

The inspection machine will be able to provide the production report, which indicates the percentage of discarded tubes and the estimate of the defective tubes (false negatives with respect to the quality criteria) that may be present in a production batch. The estimation can be made by furnishing the certification of the discarding system, namely the statistical certification for all the types of tubes made. Certification is provided via the functionality of “verification and quality certification” present in the inspection system. It consists of setting aside a few tubes (300 in our case) and experts manually verifying the defect data provided by the machine.

## 7. Conclusions

During the production of glass tubes for pharmaceutical applications, a machine vision-based inspection system can be utilized to check the quality of tubes. The need to improve accuracy in detection and advances in the process determine the need for defect detection techniques with reduced processing time. Nevertheless, specific factors that characterize the production process prevent the efficient exploiting of solutions, which are proposed in the literature.

In this work, after analyzing the characteristics of the most significant defects for the application area, we have derived the Sigma algorithm that does not change the detection quality compared to the state-of-the-art proposal, but does determine a drastic reduction in the processing time of a frame. The algorithm only performs columns level processing to detect blobs, by applying an adaptive threshold based on the Sigma Rule. In addition, it only performs row level processing to detect air lines, by applying a threshold to the variation of luminous intensity along a row. Therefore, this limits the detection effects due to the tube’s curvature, as well as the rotation and vibration of the tube, which characterize the production of the glass tube. The algorithm has been compared, in terms of detection quality and performance, with state-of-the-art solutions. The results demonstrate that, using the algorithm proposed, the processing time of the detection phase is reduced by 86%, with an increase in throughput of 268%, achieving greater accuracy in detection. The results show that the algorithm is suitable for faster production lines as it can sustain the increased sampling rates of the cameras, thus keeping the quality of detection constant or increasing it.

Moreover, the performance has been verified in real operating conditions. We carried out a stress test on two different sized batches of tubes, by setting considerably more restrictive discard conditions than the real ones. The results obtained in terms of precision and recall demonstrate the effectiveness of the technique. The processing times observed during stress testing and the actual operation are in line with what was estimated using the datasets, confirming the validity of the estimates obtained. With the reject criteria commonly adopted in production, the precision and recall values are even higher than observed in the stress test. By analyzing the effects of defect on the column on which they are located, we developed a tuning procedure that permits the determination of the algorithm’s parameters in the change of the batch of production. Furthermore, we discussed the implementation issues of the system.

## Figures and Tables

**Table 1:** Image acquisition subsystem.

System Component	Adopted Hardware
Linear Camera	Basler Racer [56]
Illuminator	Red light COBRA Slim LED Line [57]
Frame Grabber	Matrox Solios eCL/XCL-B [58] (2K)

**Table 2:** Algorithms considered in the evaluation.

Experiment Name	Pre-Processing	Defect Detection	Parameters	Post-Processing
Canny	ROI identification [14]	Canny Algorithm [19]	Hysteresis Thresholds35, 80	Class. of containers (Section 4.4)
Sigma	ROI identification [14]	Local and Global Threshold (Section 5)	k_c_ = 4.91 k_r_ = 12	Class. of containers (Section 4.4)
Niblack	ROI identification [14]	Niblack Algorithm [32]	N = 20 × 20 K = −1.7	Class. of containers (Section 4.4)

**Table 3:** Defects/defective frames and classification.

		Blobs	Air lines	Defective Frames
Expected value	TP	10	6	13
Canny	TP/FP (FN)	10/5 (0)	6/0 (0)	13/2 (0)
Sigma	TP/FP (FN)	10/3 (0)	6/0 (0)	13/1 (0)
Niblack	TP/FP (FN)	10/11 (0)	6/0 (0)	13/4 (0)

**Table 4:** Area in pixels of blob defects.

	Expected Value	Canny Algorithm	Sigma	Niblack
Cumulative Sum	1796	3293	2016	664
Cumulative Percentage	100	183.35	111.38	36.69
Avg Abs Error (%)	0	167.27	22.86	44.38

**Table 5:** Length in pixels of air line defects.

	Expected Value	Canny Algorithm	Sigma	Niblack
Cumulative Sum	3025	2552	2691	2586
Cumulative Percentage	100	84.36	88.96	85.49
Avg Abs Error (%)	0	15.42	12.58	15.60

**Table 6:** Processing time and throughput.

	Processing Time				Throughput
Algorithm	ROI	Detection	Classification	Total	FPS
Canny	7.845	61.538	9.395	89.824	11.1
Sigma	7.698	8.585	8.192	33.514	29.8
Niblack	7.934	2323.891	50.606	2642.169	0.4

**Table 7:** Processing time and throughput.

		Processing Time			Throughput	
Algorithm		ROI	Detection	Classification	Total	FPS
Canny	Without DSDRR	7.845	61.538	9.395	89.824	11.1
	DSDRRD	10.331	10.437	2.554	29.745	33.6
Sigma	Without DSDRR	7.698	8.585	8.192	33.514	29.8
	DSDRRD	10.321	1.485	2.469	17.133	58.3

**Table 8:** Stress test.

Caliber (mm)	# Tubes	Tube Accepted	Tube Discarded	Tube Validated	Tube Invalidated	TubeFp	TubeFn	P	R
8.65/.9	300	238	62	240	60	3	2	0.950	0.967
11.6/.9	300	257	43	257	43	2	2	0.953	0.953

## Data Availability

The data presented in this study are available on request from the corresponding author. Restrictions exist as data are gathered in real production processes.
